# Peroxisomal catalase deficiency modulates yeast lifespan depending on growth conditions

**DOI:** 10.18632/aging.100519

**Published:** 2013-01-19

**Authors:** Adam Kawałek, Sophie D. Lefevre, Marten Veenhuis, Ida J. van der Klei

**Affiliations:** Molecular Cell Biology, Groningen Biomolecular Sciences and Biotechnology Institute (GBB), University of Groningen; Kluyver Centre for Genomics of Industrial Fermentation, 9700CC Groningen, The Netherlands

**Keywords:** Hansenula polymorpha, peroxisome, chronological lifespan, catalase, aging, cytochrome c peroxidase

## Abstract

We studied the role of peroxisomal catalase in chronological aging of the yeastHansenula polymorpha in relation to various growth substrates. Catalase-deficient (cat) cells showed a similar chronological life span (CLS) relative to the wild-type control upon growth on carbon and nitrogen sources that are not oxidized by peroxisomal enzymes. However, when media contained methylamine, which is oxidized by peroxisomal amine oxidase, the CLS of cat cells was significantly reduced. Conversely, the CLS of cat cells was enhanced relative to the wild-type control, when cells were grown on methanol, which is oxidized by peroxisomal alcohol oxidase. At these conditions strongly enhanced ROS levels were observed during the exponential growth phase of cat cells. This was paralleled by activation of the transcription factor Yap1, as well as an increase in the levels of the antioxidant enzymes cytochrome c peroxidase and superoxide dismutase. Upon deletion of the genes encoding Yap1 or cytochrome c peroxidase, the CLS extension of cat cells on methanol was abolished. These findings reveal for the first time an important role of enhanced cytochrome c peroxidase levels in yeast CLS extension.

## INTRODUCTION

Aging is defined as progressive deterioration of cellular components resulting in loss of function and cell death. Reactive oxygen species (ROS) are considered to play a pivotal role in this process. Until recently ROS were assumed to represent toxic by-products of cellular metabolism, which inflict damage to important macromolecules such as DNA, lipids and proteins [1, 2]. Indeed, when cells age oxidative stress increases paralleled by the accumulation of oxidatively damaged macromolecules. However, recent findings indicate that this cannot fully explain age associated functional losses. Instead, ROS were shown to be crucial in several important cellular processes such as signal transduction, gene regulation, and redox regulation [3-5]. As a consequence, their complete elimination would be harmful to cells. These observations resulted in an alternative hypothesis, designated “the redox stress hypothesis”, which proposes that age-associated functional losses are caused by progressive oxidation of redox-sensitive protein thiols and consequent disruption of the redox-regulated signaling mechanisms [6].

Since depolarized mitochondria are a main source of ROS, homeostasis of this organelle is considered as a major determinant of lifespan [7-9]. A second important class of oxidative organelles includes peroxisomes, which contain hydrogen peroxide producing oxidoreductases in conjunction with antioxidant enzymes [10, 11]. Their role in aging has only recently been established [12-14].

Catalase is an important conserved peroxisomal H_2_O_2_ scavenging enzyme. Studies in human fibroblasts indicated that during aging import of peroxisomal catalase is compromised, associated with enhanced intracellular H_2_O_2_ levels, indicative for a function in cellular ROS homeostasis [15, 16]. Chemical inactivation of peroxisomal catalase accelerated aging in mammalian cells [17]. Moreover, peroxisomal catalase deficiency was shown to influence the mitochondrial redox balance [18], which furthermore implicates a role of peroxisomal catalase in aging.

*Saccharomyces cerevisiae* is widely used as a model organism to study the molecular mechanisms of aging [19]. In contrast to mammals, this yeast species contains two catalase genes encoding peroxisomal catalase A (Cta1) and cytosolic catalase T (Ctt1), respectively [20, 21]. Deletion of *CTA1* was shown to cause a decrease in the chronological lifespan (CLS) [22]. However, in another study deletion of *CTA1* caused an increase in CLS. This was explained by the elevation of H_2_O_2_ levels, which triggered expression of superoxide dismutase (*SOD2*) thereby decreasing the level of superoxide anions [23]. Moreover, overexpression of *CTA1* was shown to decrease the CLS of *S. cerevisiae*. Both observations would be in line with the redox stress hypothesis.

To better understand the role of peroxisomal catalase in aging, we used the yeast *Hansenula polymorpha* as a model organism. Akin to mammalian cells, this organism has only one catalase, which is peroxisomal [24, 25]. In contrast to *S. cerevisiae* which contains only one peroxisomal oxidase, acyl CoA oxidase, *H. polymorpha* peroxisomes contain in addition multiple other oxidases, like in mammals.

In this study we analyzed the CLS of wild-type (WT) and catalase-deficient (*cat*)*H. polymorpha* cells upon cultivation on media containing different carbon and nitrogen sources that do or do not involve peroxisome function. During growth on glucose or glycerol as carbon source in the presence of ammonium sulfate as nitrogen source peroxisomal enzymes are not required for the metabolism of the primary carbon and nitrogen sources. However, when media contain methylamine as sole nitrogen source or methanol as carbon source, the peroxisomal oxidases amine oxidase (AMO) and alcohol oxidase (AO) are required for growth. Our data indicate that the effects of the absence of peroxisomal catalase on the CLS is highly variable (ranging from a negative to a positive effect) and depends on the growth substrates.

## RESULTS

### Peroxisomal metabolism triggers lifespan changes in catalase deficient *H. polymorpha*

**Figure 1:**
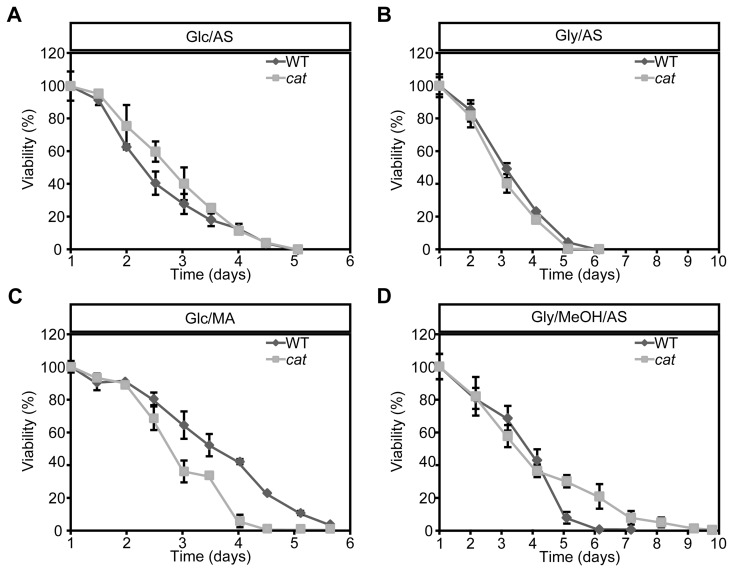
Chronological lifespan of *H. polymorpha* WT and *cat* cells grown on different carbon and nitrogen sources Cells were grown on media containing Glc/AS **(A)**, Gly/AS **(B)**, Glc/MA **(C)** or Gly/MeOH/AS **(D)**. Data represent mean ± SD, n = 3.

To analyze the role of peroxisomal catalase in the CLS of *H. polymorpha*, CLS measurements were performed using WT and *cat* strain grown on different carbon and nitrogen sources. Survival measurements started (T = 0 h) when the cultures had entered the stationary phase (Fig. S1ABCD). Very similar CLS curves and mean and maximal lifespans were observed for WT and *cat* culture grown on glucose/ammonium sulfate (Glc/AS) or glycerol/ammonium sulfate (Gly/AS) (Fig. 1AB, Table 1).

**Table 1:** Mean and maximum lifespan of *H. polymorpha* WT and *cat* cells upon growth on different carbon and nitrogen sources

Growth conditions	Strain	Mean lifespan (days)	Max. lifespan (days)
Glc/AS	WT	2.33 ± 0.06*	3.92 ± 0.35
	*cat*	2.83 ± 0.18*	4.04 ± 0.18
Gly/AS	WT	3.71 ± 0.40	5.58 ± 0.36
	*cat*	3.40 ± 0.50	5.00 ± 0.12
Glc/MA	WT	3.97 ± 0.29*	5.46 ± 0.34*
	*cat*	3.04 ± 0.51*	4.03 ± 0.30*
Gly/MeOH/AS	WT	3.81 ± 0.32	5.10 ± 0.09*
	*cat*	3.38 ± 0.41	6.77 ± 0.56*

The mean lifespan was calculated as time point when cultures reached 50% survival; the maximum lifespan was calculated as time point when cultures reached 10% survival. The data represents mean ± SD from at least 6 independent cultures.

* P<0.05 in student T test. Comparison between WT and *cat* cells in same growth conditions

When we used glucose/methylamine media (Glc/MA), *cat*cultures showed a significant reduction in both median and maximum lifespan compared to the WT control (Fig. 1C, Table 1). This reduction was not related to growth differences, as the doubling times and growth yields of both cultures were identical (Fig. S1B).

Upon growth on glycerol/methanol/ammonium sulfate (Gly/MeOH/AS) the median lifespans were similar, but the maximum lifespan of *cat*cultures was significantly enhanced relative to the WT control (Fig. 1D, Table 1). This effect was not related to differences in methanol consumption in both cultures (Fig. S1E). The final optical density of the *cat* culture on Gly/MeOH/AS was however lower relative to that of WT culture, as reported previously [26] (Fig. S1D).

### Catalase deletion affects intracellular ROS levels

**Figure 2:**
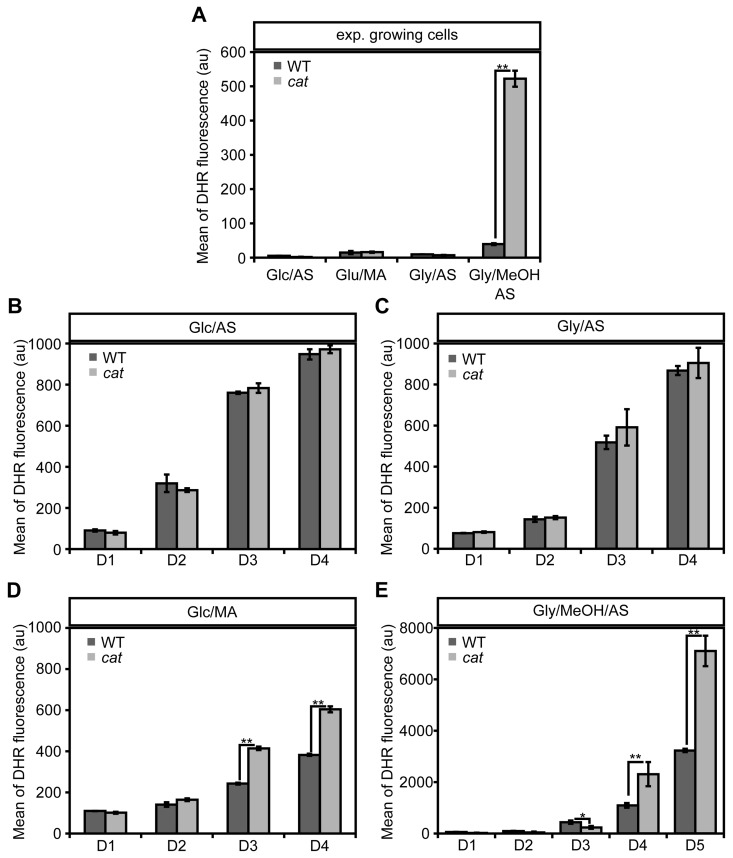
Changes in ROS levels in WT and*cat* cells during exponential growth and chronological aging DHR staining and FACS analysis was performed with exponentially growing cells (4 h after inoculation for Glc/AS, Glc/MA, Gly/AS and 16 h for Gly/MeOH/AS) **(A)** and chronologically aging cells grown on media containing Glc/AS **(B)**, Gly/AS **(C)**, Glc/MA **(D)** or Gly/MeOH/AS **(E)**. Data represents mean ± SD, n = 3. * - P<0.05, ** - P<0.01 in student T test.

To check the impact of *CAT* deletion on ROS levels, we determined the levels of these compounds using the fluorescent dye dihydrorodhamine 123 (DHR) and FACS (Fig. 2). As shown in Figure 2A cells in the exponential growth phase on Glc/AS, Gly/AS and Glc/MA showed very similar, low ROS levels. Upon growth of WT cells on Gly/MeOH/AS ROS levels were slightly increased. However, in *cat* cells grown on Gly/MeOH/AS ROS levels were strongly enhanced relatively to that in *cat* cells grown on the other substrates as well as compared to the WT control grown on Gly/MeOH/AS (>13 ×).

During chronological aging ROS levels increased in all cultures with time. No differences in ROS levels were observed during chronological aging of Glc/AS or Gly/AS grown WT and *cat* cultures (Fig. 2BC). However, in *cat* cultures grown on Glc/MA and Gly/MeOH/AS ROS levels increased to higher levels compared to the WT controls (Fig 2DE).

In the Gly/MeOH/AS culture the ROS levels were lower at day 1 (40 h after inoculation; Fig. 2E) relative to the levels observed in the exponential cultures (16 h after inoculation; Fig. 2A) most likely related to the fact that in the stationary phasemethanol oxidation had ceased (Fig. S1E).

### Activation of stress adaptation pathways

The high ROS levels observed during the exponential growth phase of Gly/MeOH/AS *cat* cultures may have induced stress response genes, which are often linked to lifespan extension [27-29]. To test this we analyzed the resistance of WT and *cat* cells to externally added H_2_O_2_ or acrolein. Acrolein is a toxic byproduct of lipid peroxidation, which occurs in living cells under conditions of oxidative stress.

**Figure 3:**
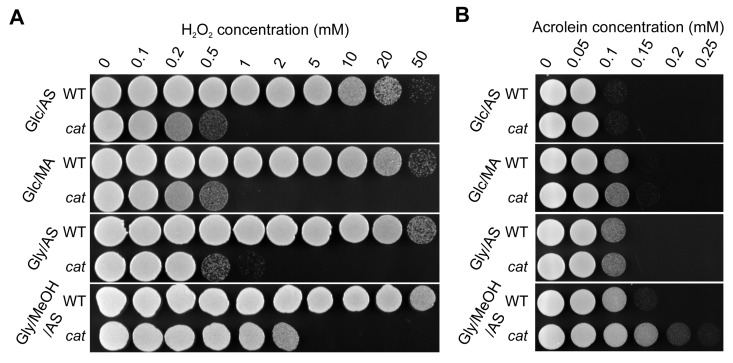
H_2_O_2_ and acrolein resistance of stationary phase WT and *cat* cells grown on different media Cells were grown to the stationary phase (16 h on Glc/AS, Glc/MA or Gly/AS and for 40 h on Gly/MeOH/AS). Equal amounts of cells were treated with increasing concentrations of H_2_O_2_**(A)** or acrolein **(B)**. Experiments were repeated at least 3 times. Representative plates are shown.

Independent of the cultivation conditions used, *cat* cells displayed decreased resistance to externally added H_2_O_2_ in comparison to the WT (Fig. 3A), which most likely can be fully explained by the absence of catalase activity in these cells. However, *cat* cells grown on Gly/MeOH/AS were more resistant to H_2_O_2_ than Glc/AS, Glc/MA or Gly/AS grown *cat* cells, suggesting that a catalase independent H_2_O_2_ defense pathway is induced in these cells. Both WT and *cat* cultures showed lowest acrolein resistance upon growth on Glc/AS, whereas the resistance slightly increased for both strains upon growth on Glc/MA or Gly/AS, and further increased upon growth on Gly/MeOH/AS. Interestingly, *cat* cells grown on Gly/MeOH/AS showed higher acrolein resistance than WT cells (Fig. 3B).

### Identification of *H. polymorpha* Yap1

In *S. cerevisiae* adaptation to H_2_O_2_ and thiol reacting compounds, such as acrolein, is mediated by the basic leucine-zipper transcription factor Yap1 [30, 31]. We therefore analyzed the role of *H. polymorpha* Yap1. The *H. polymorpha YAP1* gene was identified using BLAST searches in the *H. polymorpha* genome database using Yap1 sequences from *P. pastoris*[32], *S. pombe*[33] and *S. cerevisiae*[34]. The putative *H. polymorpha* Yap1 homologue showed 35% sequence identity to *P. pastoris* Yap1, 25% to *S. pombe* Yap1 and 24% to *S. cerevisiae* Yap1. *YAP1* (accession number EFW96135) encodes a protein of 420 amino acids containing a predicted nuclear localization signal (NLS) [35] at the N-terminus (amino acids 30-50) and a leucine rich nuclear export signal (NES) [36] at the C terminus (amino acids 386-392).

Moreover, it contains 6 cysteines, two of which are present within the NES. Sequence alignments indicated that those features are conserved (Fig. S2).

### Yap1 is involved in H_2_O_2_ and acrolein resistance of *H. polymorpha cat* cells

**Figure 4:**
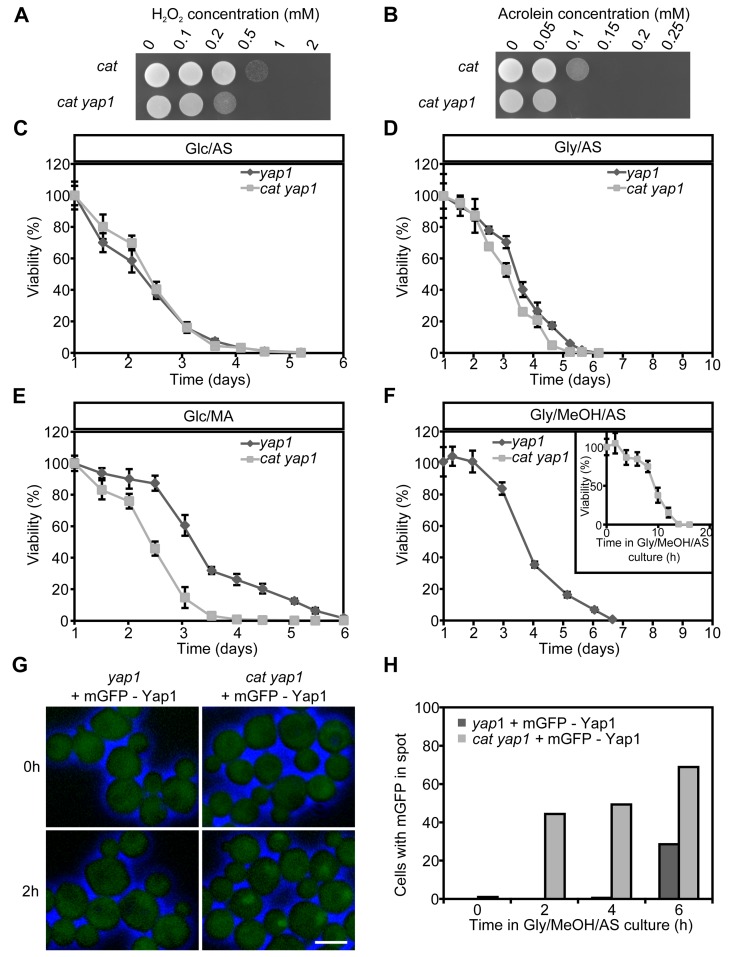
Role of Yap1 in H_2_O_2_ and acrolein resistance and CLS extension Expotentially Glc/AS growing *cat* and *cat yap1* cells were challenged with increasing concentrations of H_2_O_2_ **(A)** or acrolein **(B)**. Experiments were repeated twice and a representative experiment is shown. CLS curves of *yap1* and *cat yap1* strains grown on Glc/AS **(C)**, Gly/AS **(D)**, Glc/MA **(E)** or Gly/MeOH/MA **(F)**. Number of colonies obtained after 16 h was set to 100% viability except for *cat yap1* cells for which the initial viability was measured immediately after shifting to Gly/MeOH medium (inset). Data represents mean viability ± SD, n = 3. **(G)** Fluorescence microscopy of mGFP-Yap1 complemented *yap1* and *cat yap1* strains before (0 h) and two hours after the shift to Gly/MeOH/AS media. Brightfield images were false-colored into blue to mark cell borders. Bar - 3μm. **(H)** Percentage of cells showing mGFP-Yap1 concentrated in a spot after shifting mGFP-Yap1 producing *yap1* and *cat yap1* strains to Gly/MeOH/AS.

We first analyzed the role of Yap1 in stress resistance using *yap1* and *cat yap1* deletion strains. These strains showed similar growth profiles as *cat* and WT cultures (compare Fig. S1) in media containing Glc/AS, Glc/MA or Gly/AS (Fig. S3ABC). In Gly/MeOH/AS medium, *cat yap1* cells showed a slightly enhanced doubling time and a reduced yield relative to *cat* cultures (Fig. S3D; compare Fig. S1D). Analysis of the resistance to H_2_O_2_ and acrolein, revealed that this was reduced in *cat yap1* cells relative to the *cat* controls (Fig. 4AB).

### Yap1 is required for lifespan extension of Gly/MeOH/AS grown *cat* cells

Next, we tested whether Yap1 plays a role in CLS determination. No strong differences in CLS between *yap1* and *cat yap1* cultures were observed upon growth on Glc/AS or Gly/AS (Fig. 4CD). Moreover, the curves were similar to those observed for WT and *cat* (Fig. 1). On Glc/MA, the *yap1* curve resembled that of WT, whereas the *cat1 yap1* survival curve was similar to that of *cat* cells (Fig. 4E, compare Fig. 1C), indicating that Yap1 is not an important player in determining the CLS at these conditions. The CLS of *yap1* cultures on Gly/MeOH/AS was comparable to that of the WT control (Fig. 4F, compare Fig. 1D). In contrast a very rapid decrease in survival was observed for Gly/MeOH/AS grown *cat yap1* cells resulting in less than 10% survival within 12 h after shifting the cells to this medium (Fig. 4F, inset). These data indicate that Yap1 is of major importance for survival of Gly/MeOH/AS grown *cat* cultures.

To seek further evidence for the function of Yap1, we performed localization experiments using a gene encoding an N-terminal fusion of mGFP with Yap1 under control of the *YAP1* promoter. Growth and CLS experiments revealed that the fusion protein fully complemented the *yap1* deletion strain (data not shown). Upon growth on Glc/AS, Glc/MA or Gly/AS, mGFP-Yap1 was predominantly localized to the cytosol in WT and *cat* cells (data not shown). However, after the shift of Glc/AS-grown cells to Gly/MeOH/AS, mGFP-Yap1 rapidly migrated (within two hours) to the nucleus of *cat* cells, but not of WT cells (Fig. 4GH). These data reveal rapid Yap1 activation upon exposure of *cat* cells to methanol.

### Cytochrome c peroxidase and superoxide dismutase, but not glutathione reductase, are induced in Gly/MeOH/AS grown *cat* cells

**Figure 5:**
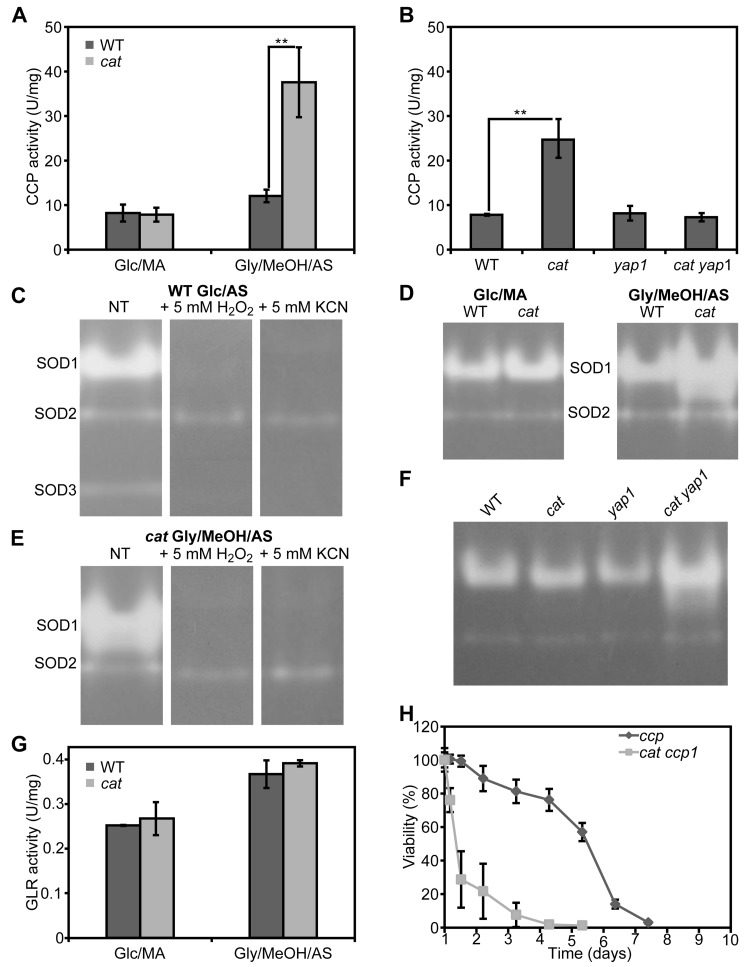
Cytochrome c peroxidase, superoxide dismutase and glutathione reductase activities in *H. polymorpha* WT and *cat* cells grown on different media **(A)** Measurements of CCP activities in crude extracts of WT and *cat* grown on Glc/MA and Gly/MeOH/AS for 16 h. **(B)** Measurements of CCP activities in crude extracts of WT, *cat*, *yap1* and *cat yap1* cells grown on Gly/MeOH/AS for 4 h. **(C)** Superoxide dismutase isozyme profiling in WT cells. SOD activity was detected in crude extracts prepared from cells grown for 16 h on Glc/AS without pre-treatment (NT) or upon 30 min pre-incubation with 5 mM H_2_O_2_ or 5 mM KCN. **(D)** SOD activity in WT and *cat* cells grown for 16 h on Glc/MA or Gly/MeOH/AS. **(E)** SOD activity of in *cat* cells grown for 16 h in Gly/MeOH/AS detected without pre-treatment (NT) or after 30 min pre-incubation with 5 mM H_2_O_2_ or 5 mM KCN. **(F)** SOD activities in WT, *cat*, *yap1*, *cat yap1* cells grown for 4 h on Gly/MeOH/AS. **(G)** Glutathione reductase activities in WT and *cat* cells grown in Glc/MA or Gly/MeOH/AS for 16 h. **(H)** CLS of *ccp* and *cat ccp* cells in Gly/MeOH/AS medium. Viability experiments were started 12 h after inoculation of the media. Experiments were repeated at least twice. Data represent mean activities ± SD, n = 3, ** - p<0.01. For native gels, representative gels are shown.

Induction of antioxidant enzymes has been implicated in yeast lifespan extension. We determined the activities of cytochrome c peroxidase (CCP), superoxide dismutase (SOD) and glutathione reductase (GLR) in crude extracts of WT and *cat* cells grown for 16 h on Glc/MA or Gly/MeOH/AS. As shown in Figure 5A, CCP is induced 3-fold in Gly/MeOH/AS-grown *cat* cells relative to the WT control. During growth on Glc/MA CCP activities were similar in both strains (Fig. 5A).

To check whether induction of CCP depends on Yap1, we also measured CCP activities in Gly/MeOH/AS grown*yap1* and *cat yap1* cells, using WT and *cat* cells as controls. Deletion of *YAP1* alone did not affect activity of this enzyme relative to WT. CCP activity was enhanced in *cat* cells but not in *cat yap1* cells relative to the WT control (Fig. 5B).

SOD isozyme profiling of stationary WT cells grown on Glc/AS medium revealed the presence of 3 bands(Fig. 5C). The bands of lowest (designated SOD1) and highest (designated SOD3) relative mobility were inactivated by H_2_O_2_ and KCN and therefore represent Cu/Zn containing SOD enzymes. The middle band (designated SOD2) is most likely a Mn containing SOD, as it appeared to be resistant to both compounds.

Comparison of isozyme profiles of WT and *cat* cells grown on Glc/MA or Gly/MeOH/AS, revealed that *cat* cells grown on Gly/MeOH/AS showed the highest SOD1 activity (Fig. 5D), which was inhibited by H_2_O_2_ and KCN (Fig. 5E).

Because *cat yap1* cells very rapidly die on Gly/MeOH/AS media (Fig. 4F), SOD activities were determined in these cultures obtained 4 hours after the shift from Glc/AS to Gly/MeOH/AS. At this time point the activity of SOD1 had not yet increased in *cat* cells. In *cat yap1* cells however SOD levels were increased (Fig. 5F), suggesting that SOD1 expression is not controlled by Yap1.

Enzyme activities of GLR were similar in WT and *cat* cells grown on Glc/MA or Gly/MeOH/AS (Fig 5E).

Together our data indicate that in *cat* cells grown on Gly/MeOH/AS, but not on Glc/MA, genes are expressed that enhance stress resistance and extend the CLS. One of these genes encodes CCP and is induced in a Yap1 dependent way, another one is SOD1, which is not under control of Yap1.

### CCP plays a role in lifespan extension of *cat* cells grown on Gly/MeOH/AS

The increase in lifespan of cat cells on Gly/MeOH/AS depends on Yap1 and is paralleled by an increase in CCP activity, which also depends on Yap1. To test whether indeed enhanced CCP levels are essential for lifespan extension, CLS experiments were performed with a *ccp* deletion strain and a *cat ccp* double deletion strain. As shown in Figure 5H, CCP is essential for survival of*cat* cells in Gly/MeOH/AS medium. Deletion of *CCP* alone did not have a significant effect on CLS (Fig. 5H). These data suggest that in *cat* cells in Gly/MeOH/AS medium Yap1 mediated induction of CCP, is essential for lifespan extension.

### AMO activity is partially reduced in Glc/MA grown *cat* cells

**Figure 6:**
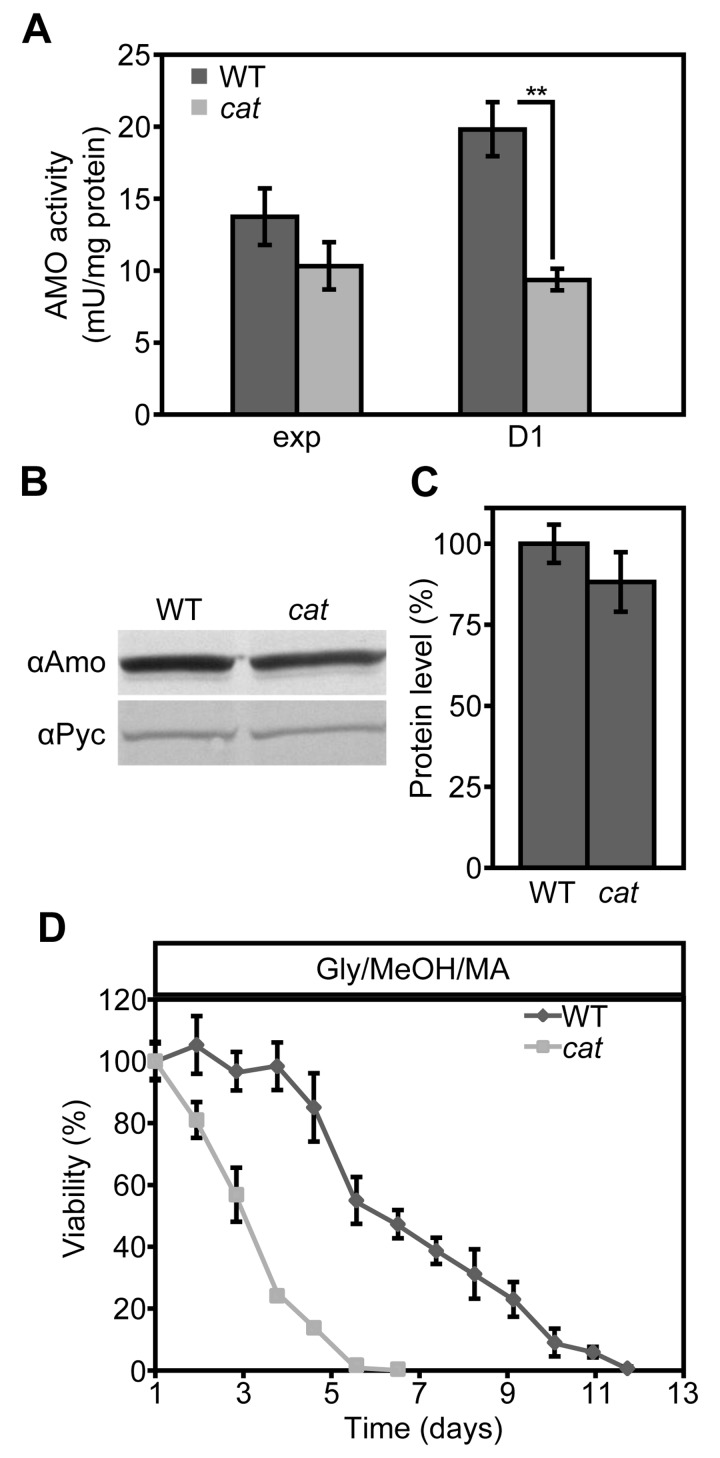
AMO activities are reduced in Glc/MA *cat* cells relative to the WT control **(A)** Measurements of AMO activities in crude extracts of WT and *cat* cells grown on Glc/MA. Activity is expressed as mU/mg protein. **(B)** Western blot analysis of AMO protein levels in cells grown for 16 h on Glc/MA. Cytosolic pyruvate decarboxylase Pyc1 was used as a loading control. **(C)** Quantification of AMO protein relative to Pyc1 of the blot shown in B. Data represent mean ± SD, n = 3. **(D)** CLS of WT and *cat* cells grown on Gly/MeOH/MA. Time point 0 h indicates time when cells were shifted to final medium. The number of colonies obtained after 16 h of growth was set to 100% viability. Experiments were repeated twice. Data represents mean ± SD, n = 3.

We recently showed that the presence of MA instead of AS as sole nitrogen source in *H. polymorpha* cultures extends the CLS, because MA serves as extra energy source in stationary phase cultures [37]. Since the lifespan of *cat* cells in Glc/MA is reduced relative to the WT control, but similar to that of Glc/AS cultures (Fig. 1AC) we investigated whether this is related to the absence of amine oxidase (AMO) activities, which would prevent the generation of energy from MA during chronological aging. As shown in Figure 6, AMO activity was still present albeit reduced by 53% in *cat* cells in comparison to WT in stationary cultures (Fig. 6A). However, the residual AMO activity most likely still allows to metabolize MA during the stationary phase. Western blot analysis revealed that AMO protein levels were only slightly reduced, indicating that part of the AMO enzyme is inactivated in*cat* cells (Fig. 6BC).

### The activation of stress responsive genes does not alleviate the negative effect of MA on the CLS of *cat* cells

Our data suggested that upon growth on Gly/MeOH/AS, but not on Glc/MA, resistance genes are induced in *cat* cells. To test whether the induction of these genes can overcome the negative effect of MA on cell survival, we compared the CLS of WT and *cat* cells grown on Gly/MeOH supplemented with MA as sole nitrogen source. In agreement with our previous data addition of MA extended the lifespan of WT cells, however *cat* cultures showed a strongly reduced mean and maximum lifespan relative to the WT control (Fig. 6D)

## DISCUSSION

In yeast two types of aging have been defined, namely chronological and replicative aging. The chronological lifespan is the time cells can survive in a non-dividing state, whereas the replicative lifespan represent the number of buds a mother cell produces before it dies. These two types of aging are regulated by partially overlapping regulatory mechanisms. Several recent studies focused on the role of acidification, which accelerates both modes of aging in yeast as well as in mammalian cells [38,39]. Here we studied the role of another important factor in aging, namely reactive oxygen species (ROS).

We have analyzed the role of catalase, a major peroxisomal antioxidant enzyme, on the chronological lifespan of *H. polymorpha*. Previously, several reports appeared on the function of this enzyme in chronological aging of *S. cerevisiae*. In one of them, deletion of *CTA1*, the gene encoding peroxisomal catalase, was shown to lead to shorter lifespan of *S. cerevisiae* upon growth on 2% glucose. [22], whereas in another study this increased the CLS [23]. A complicating factor in the analysis of the role of peroxisomal catalase in chronological aging of *S. cerevisiae* is that baker's yeast also contains a cytosolic isoenzyme, Ctt1. By contrast, the *H. polymorpha* has only one catalase gene, which encodes a peroxisomal protein, like in man.

Our data indicate that in *H. polymorpha* the effect of catalase deficiency on CLS varies with the cultivation conditions and ranges from a negative effect (Glc/MA) via no effect (Glc/AS and Gly/AS) to a positive effect (Gly/MeOH/AS). An important difference between MA and MeOH utilization is the amount of H_2_O_2_ that is generated during the growth phase. This is higher for MeOH, which is used as carbon source, relative to MA, which serves as nitrogen source (C/N ratio of *H. polymorpha* cells = 7).

During growth on Glc/AS and Gly/AS no peroxisomal enzymes are involved in the metabolism of the carbon and nitrogen source. Hence, no peroxisomal hydrogen peroxide is produced. This may explain why deletion of *CAT* has no effect on the CLS upon growth on these substrates (Fig. 1AB). Also in these media the ROS levels were similar in WT and *cat* cells, both in the exponential growth phase (Fig. 2A) and during chronological aging (Fig. 2BC).

We recently showed that growth of WT *H. polymorpha* cells on Glc/MA extends the CLS relative to Glc/AS because of the generation of NADH from formaldehyde, the MA oxidation product, during the stationary phase [37]. However, we now observed that the CLS of the *cat* cells grown on Glc/MA is reduced relative to the WT control. MA metabolism during the stationary phase results in H_2_O_2_ production. In the absence of catalase H_2_O_2_ will be decomposed by processes that require reducing equivalents, e.g. via CCP. Hence, it is likely that the net NADH gained by MA metabolism is lost due to NADH requiring H_2_O_2_ degradation in *cat* cells resulting in median and maximal lifespans similar to that observed for Glc/AS grown *cat* cells.

The extended CLS of the Gly/MeOH/AS-grown *cat* strain was related to the presence of MeOH in the media during the growth phase, because the CLS of *cat* and WT cells grown on Gly/AS was identical. A positive effect of NADH generation from formaldehyde generated from methanol oxidation during chronological aging can be ruled out as methanol was consumed at the initiation of the CLS experiment (Fig. S1E).

Our data suggest that enhanced ROS levels during the exponential growth phase on Gly/MeOH/AS (Fig. 2A) is beneficial and trigger the induction of stress response genes [23]. In line with this, Gly/MeOH/AS grown *cat* cells showed enhanced resistance towards H_2_O_2_ and acrolein relative to Gly/AS grown *cat* cells. Indeed enzyme activity measurements revealed the induction of CCP and SOD. The enhanced resistance against acrolein may be explained by inducting of old yellow enzyme (OYE) [40].

Yap1 has been described as important transcription factor in regulating stress responsive genes in different yeast species and Yap1 binding sites have been identified in promoter regions of genes involved in ROS scavenging. Using the Yeastract tool [41] we analyzed promoter regions of candidate *H. polymorpha* genes for putative Yap1 binding sites [34,42,43] (Table 2). These motifs were indeed identified in *H. polymorpha* genes encoding CCP, SOD and OYE (*HYE1*, *HYE3*), but not in Cu/Zn SOD (Table 2). In line with this is the observation that CCP is not up-regulated in a *cat yap1* double deletion strain (Fig. 5B), confirming the role of Yap1 in CCP induction. Also, acrolein resistance was impaired in the double deletion strain (Fig. 4B), pointing to a role for Yap1 in OYE induction. As expected SOD induction was not affected by *YAP1* deletion (Fig. 5F).

**Table 2:** Putative Yap1 binding sites in selected *H. polymorpha* genes encoding homologues of *S. cerevisiae* proteins related to ROS scavenging and acrolein resistance

Enzyme	*S. cerevisiae*^1^	*H. polymorpha*^2^	Yap1 binding site^3^
Cu/Zn superoxide dismutase	*SOD1 (YJR104C)*	50222	-
cytochrome c peroxidase	*CCP1 (YKR066C)*	51133	-56
FMN oxidoreductase Hye1	*OYE3 (YPL171C)*	49662	-52
FMN oxidreductase Hye3	*-*	59065	-44 -55

1 Names of *S. cerevisiae* enzymes implicated in oxidative stress response including their accession numbers.

2 Protein ID of identified *H. polymorpha* homologue in genome sequence of strain NCYC495 leu1.1 v2.0 (http://genome.jgi.doe.gov/Hanpo2/Hanpo2.home.html)

3 Position of putative Yap1 binding site upstream of the start codon.

The important role of CCP in CLS extension of *cat* cells was demonstrated by the observation that *cat ccp* cells, but not *ccp* cells, have a very short CLS on Gly/MeOH/AS (Fig. 5G).

Summarizing, our findings indicate that upon growth of*cat* cells on Gly/MeOH/AS, but not on Glu/MA, several stress responsive genes are induced, among others CCP, SOD and OYE, of which CCP and OYE induction is Yap1 dependent. Yap1 activation is most likely caused by the high ROS levels during the exponential growth on Gly/MeOH/AS, which is not observed in Glu/MA cells. Consistent with this is the observed migration of Yap1 to the nucleus upon transfer of glucose-grown *cat* cells to these media.

Finally, we show for the first time the important role of CCP in CLS extension as no CLS extension was observed in *cat ccp1* cells grown on Gly/MeOH/AS. This makes CCP another important player in yeast CLS next to SOD.

Taken together, we have shown that peroxisomal catalase is important in regulating lifespan. However, the actual effect strongly depends on the carbon and nitrogen sources used for growth.

## METHODS

### Strains and growth conditions

The *H. polymorpha* strains used in this study are listed in Table 3. Cells were grown on mineral medium (MM) [44] supplemented with different carbon sources: 0.25% glucose (Glc), 0.05% glycerol (Gly) or a mixture of 0.05% glycerol and 0.5% methanol (Gly/MeOH), and nitrogen sources: 0.25% ammonium sulfate (AS) or 0.25% methylamine(MA). 6 mM K_2_SO_4_ was added when methylamine was used as nitrogen source. When required, leucine was added to a final concentration of 60 μg/ml. Selection of yeast transformants was performed on YND plates (0.17% Yeast Nitrogen Base w/o AA, w/o N, 0.25% NH_4_SO_4_, 1% glucose and 2% agar) or on YPD (1% yeast extract, 1% glucose, 1% peptone, 2% agar) supplemented with 300 μg/ml hygromycin B (Sigma) or 100 μg/ml nourseothricin. For viability determination cells were plated on YPD agar plates. For cloning purposes, *E. coli* DH5α or GM48 were used. Bacteria were grown at 37°C in LB media supplemented with 100 μg/ml ampicillin or 50 μg/ml kanamycin when required.

**Table 3:** Strains used in this study

Strain	Description	Origin
WT *ura3*	NCYC495 *ura3 leu1.1*	[53]
WT	NCYC495 *leu1.1*	this study
*cat*	NCYC495 *CAT::URA3 leu1.1*	this study
*yap1*	NCYC495 *YAP1::HPH leu1.1*	this study
*cat yap1*	NCYC495 *CAT::URA3 YAP1::HPH leu1.1*	this study
*yap1 + mGFPYAP1*	*yap1* pEXP_P_*YAP1*__mGFP-*YAP1*_T_*AMO*_	this study
*cat yap1 + mGFP_YAP1*	*cat yap1* pEXP_P_*YAP1*__mGFP-*YAP1*_T_*AMO*_	this study
*ccp*	NCYC495 *CCP::HPH leu1.1*	this study
*cat ccp*	NCYC495 *CAT::URA3 CCP::HPH leu1.1*	this study

### Cloning and construction of yeast strains

The plasmids and primers used in this study are listed in Tables 4 and 5. All cloning was performed using Gateway technology (Invitrogen). Standard recombinant DNA techniques and transformation of *H. polymorpha* was performed as described previously [45]. All deletions and integrations were confirmed by PCR and southern blotting.

**Table 4:** Plasmids used in this study

Plasmid	Description	Origin
pDONR_41	Standard Gateway vector	Invitrogen
pDONR_221	Standard Gateway vector	Invitrogen
pDONR_23	Standard Gateway vector	Invitrogen
pDEST_43	Standard Gateway vector	Invitrogen
pENTR41-5'CAT	pDONR-41 containing upstream region of *CAT* gene/kan^R^	this study
pENTR221-URA3	pDONR_221 containing *HpURA3* gene complementing uracil auxotrophy/kan^R^	[54]
pENTR23-3'CAT	pDONR-41 containing 3' region of *CAT*/kan^R^	this study
pDEL_*CAT*_URA3	pDEST43 containing deletion cassette 5'*CAT* - URA3 - 3'*CAT*/amp^R^	this study
pENTR23_T_*AMO*_	pDONR-23 containing *AMO* terminator/kan^R^	[55]
pDEST_43_NAT	Gateway destination vector with the *Streptomyces noursei nat1* gene/amp^R^	[56]
pENTR41_5'YAP1	pDONR-41 containing 5' region of *YAP1* gene/kan^R^	this study
pENTR221_HPH	pDONR_221 containing HPH resistance marker/ kan^R^	[56]
pENTR23_3'YAP1	pDONR-23 containing 3' region of *YAP1* gene/kan^R^	this study
pDEL_*YAP1*_HPH	pDEST43 containing deletion casette 5'*YAP1* - HPH - 3'*YAP1*/amp^R^	this study
pHIPZ_mGFP fusinator	pHIPZ containing mGFP and *AMO* terminator/amp^R^	[56]
pENTR41_P_*YAP1*__mGFP	pENTR-41 containing *YAP1* promoter fused with mGFP/kan^R^	this study
pENTR221_*HpYAP1*	pENTR-221 containing *YAP1* gene/kan^R^	this study
pEXP_P_*YAP1*__mGFP-*HpYAP1*_T_*AMO*_	pDEST-43_NAT containing expression cassette P_*YAP1*__mGFP-*YAP1*_T_*AMO*_/amp^R^	this study
pENTR41_5'CCP	pDONR-41 containing 5' region of *CCP* gene/ kan^R^	this study
pENTR23_3'CCP	pDONR-23 containing 3' region of *CCP* gene/ kan^R^	this study
pDEL_*CCP*_HPH	pDEST43 containing deletion casette 5' *CCP* - HPH - 3' *CCP*/amp^R^	this study

**Table 5:** Oligonucleotides used in this study

Primer name	Sequence
41_5CAT_F	GGGGACAACTTTGTATAGAAAAGTTGGTTTGTGGATTTTGCTGTACCGCG
41_5CAT_R	GGGGACTGCTTTTTTGTACAAACTTGATACTTGTAGCCCGTGGAATCCAG
23_3CAT_F	GGGGACAGCTTTCTTGTACAAAGTGGAACAGGAGTCCCTTGTCAAGAACG
23_3CAT_R	GGGGACAACTTTGTATAATAAAGTT-GTGACGGTCTGCGTCCTCTTGTTAC
Catdelcas_F	GCTGATACCAGCGGATAACA
Catdelcas_R	TGTGCTGCAAGGCGATTAAG
41_5YAP1_F	GGGGACAACTTTGTATAGAAAAGTTGTTCGGAATGCGCTAATCAGTGT
41_5YAP1_R	GGGGACTGCTTTTTTGTACAAACTTGTTTAGGTAGCGTATTTAAGGGTAAGG
23_3YAP1_F	GGGGACAGCTTTCTTGTACAAAGTGGTAATCACGGGTCTTGTTGATATTG
23_3YAP1_R	GGGGACAACTTTGTATAATAAAGTTGTCGCTGCGATTATCATTTGAC
Yapdelcas_F	CGGAATGCGCTAATCAGTGT
Yapdelcas_R	CGCTGCGATTATCATTTGAC
41_Prom_Yap1F	GGGGACAACTTTGTATAGAAAAGTTGTTGACGCCGATTTGGACCAG
41_mGFP_R	GGGGACTGCTTTTTTGTACAAACTTGTTCCCTTGTACAGCTCGTCCATG
2PGFP_O_F	CCTTACCCTTAAATACGCTACCTAATAATGAGCAAGGGCGAGGAG
2PGFP_O_R	CTCCTCGCCCTTGCTCATTATTAGGTAGCGTATTTAAGGGTAAGG
221_YAP1_F	GGGGACAAGTTTGTACAAAAAAGCAGGCTTTATGTCCACAGCAACCCCAGGTG
221_YAP1_R	GGGGACCACTTTGTACAAGAAAGCTGGGTGTCAGCGATTCATCGCACGTGTG
41_5CCP_F	GGGGACAACTTTGTATAGAAAAGTTGTTTATCAGAAAGTTCCTGGACGGTA
41_5CCP_R	GGGGACTGCTTTTTTGTACAAACTTGTGATGTTCACCCCGCACAG
23_3CCP_F	GGGGACAGCTTTCTTGTACAAAGTGGTAGGTTCCAGCAAGAGCAAAAC
23_3CCP_R	GGGGACAACTTTGTATAATAAAGTTGTCGTCCAAAAGCAGCTTGAA
Ccpdelcas_F	TATCAGAAAGTTCCTGGACGGTA
Ccpdelcas_R	CGTCCAAAAGCAGCTTGAA

### Construction of a *H. polymorpha cat* deletion strain

A catalase deletion strain (*cat*) was constructed by replacing the genomic region of *CAT* (P30263) comprising nucleotides +1 to +1256 by the auxotrophic marker for uracil (*URA3*). To this end the first region -399 to 0 of the *CAT* gene was amplified using primers 41_5CAT_F and 41_5CAT_R with attB sites and recombined into pDONR41 yielding pENTR_41_5'CAT. At the same time region +1256 to +1665 was amplified using primers 23_3CAT_F and 23_3CAT_R and recombined with pDONR23 yielding pENTR_23_3'CAT.

A deletion cassette containing 5’ and 3’ fragments of the *CAT* gene and the *URA3* marker was assembled in pDEST43 with Gateway LR reaction using plasmids pENTR_41_5'CAT, pENTR_221_URA3 and pENTR_ 23_3'CAT. The resulting plasmid pDEL_CAT_URA3 was used as a template to amplify a deletion cassette of 2595 bp in PCR reaction using primers Catdelcas_F and Catdelcas_R. The purified PCR product was transformed into *H. polymorpha* NCYC 495 *ura3 leu1.1* and colonies were selected on YND with leucine.

### Construction of *H. polymorpha yap1* and *cat yap1* strains

*YAP1* (EFW96135) was deleted by replacing nucleotides -2 to +1262 by a hygromycin B resistance cassette (HPH). To this end a fragment containing region -280 to -3 from the start codon was first amplified from *H. polymorpha* genomic DNA using primers 41_YAP1_F and 41_Yap1_R and recombined in Gateway BP reaction into pDONR_41 yielding plasmid pENTR_41_5'YAP1. Similarly region +1263 to +1639 was amplified using primers 23_YAP1_F and 23_YAP1_R and recombined into pDONR_23 yielding pENTR_23_3'YAP1. Plasmids pENTR_41_5'YAP1, pENTR_221_HPH and pENTR_23_3'YAP1 were recombined in Gateway LR reaction with pDEST_43_ NAT yielding plasmid pDEL_*YAP1*_HPH. A deletion cassette of 2417bp was amplified from this plasmid using primers Yapdelcas_F and Yapdelcas_R and used for transformation of *H. polymorpha* WT and *cat* cells. Transformants were selected on YPD with hygromycin.

### Complementation of *yap1* with mGFP-*YAP1*

The *YAP1* deletion strain was complemented by insertion of an expression cassette containing the *YAP1* promoter, followed by a gene encoding the N terminally mGFP tagged Yap1 and the *AMO* terminator into the *YAP1* promoter region. To this end first the *YAP1* promoter (region -400 to 0) was amplified from genomic DNA using primers 41_Prom_Yap1F and 2PGFP_O_R. The mGFP ORF without a stop codon was amplified from the pHIPZ_mGFP fusinator plasmid using primers 2PGFP_O_F and 41_mGFP_R. PCR fragments were combined and used as a template in second overlay PCR using primers 41_Prom_Yap1F and 41_mGFP_R. The obtained DNA fragment was used in a Gateway BP reaction with pDONR_41 yielding pENTR_41_ P*_YAP1_*_mGFP. Next *YAP1* ORF was amplified from genomic DNA using primers 221_YAP1_F and 221_YAP1_R and recombined in Gateway BP reaction with pDONR_221 resulting in plasmid pENTR_221_*YAP1*. Plasmids pENTR_41_P*_YAP1_*_mGFP, pENTR_221_*YAP1*, pENTR_23_T*_AMO_* were recombined in a Gateway LR reaction with pDEST_43_NAT yielding plasmid pEXP_P*_YAP1_*_mGFP-*YAP1*_T*_AMO_*. This plasmid was transformed to *E.coli* GM48 to obtain unmethylated DNA. After linearization with XbaI plasmid DNA was transformed into *yap1* and *cat yap1* cells. Colonies were selected on YPD supplemented with nourseothricin.

### Construction of *H. polymorpha ccp* and *cat ccp* strains

*CCP* (EFW94326) was deleted by replacing nucleotides -7 to +376 by a HPH cassette. Region -280 to -3 from the start codon was first amplified from *H. polymorpha*genomic DNA using primers 41_CCP_F and 41_CCP_R and recombined in Gateway BP reaction into pDONR_41 yielding plasmid pENTR_41_5'CCP. Similarly, region +376 to +795 was amplified using primers 23_CCP_F and 23_CCP_R and recombined into pDONR_23 yielding pENTR_23_3'CCP. Plasmids pENTR_41_5'CCP, pENTR_221_HPH and pENTR_23_ 3'CCP were recombined in a Gateway LR reaction with pDEST_43_NAT yielding plasmid pDEL_*CCP*_HPH. A deletion cassette of 2633 bp was amplified from this plasmid using primers Ccpdelcas_F and Ccpdelcas_R and used for transformation of *H. polymorpha* WT and *cat* cells. Transformants were selected on YPD with hygromycin.

### Chronological lifespan measurements

Cells were extensively precultivated on media containing 0.25% glucose and 0.25% ammonium sulfate. Mid-exponential cultures (OD_600__nm_ = 1.8) cultures were diluted to an OD_600 nm_ of 0.1 in the final medium. Survival measurements were started after the cultures reached the stationary phase. This was 16 h for cultures on Glc/AS,Glc/MA and Gly/AS and 40 h for cultures grown on Gly/MeOH/AS unless stated otherwise. The number of cells per ml of culture was determined using CASY^®^ Model TT (Roche Applied Science). 500 cells were plated on YPD agar plates in triplicate. Plates were incubated at 37°C for 36 to 48 hours and photographed. Colony numbers were counted using an ImageJ plugin. The number of colony forming units at the first time point (invariably approximately 500) was set to 100%.

### Stress resistance experiments

WT and *cat* cells grown on for 16 h on 0.25% glucose / ammonium sulfate, 0.25% glucose / 0.25% methylamine, 0.05% glycerol / ammonium sulfate or 40h for 0.05% glycerol / 0.5% methanol / ammonium sulfate were collected by centrifugation and resuspended in 50mM potassium phosphate buffer pH=6.0 to an OD_600 nm_ of 0.3. 1 ml of the suspension was challenged with increasing concentrations of H_2_O_2_ or acrolein for 1 hour at 37°C. Cells were washed once and resuspended in 1 ml 50 mM potassium phosphate buffer pH=6.0. 5μl of the suspension was spotted on YPD agar plates and plates were incubated 24 h at 37°C.

### Biochemical methods

Preparation of cell extracts [46], determination of methanol concentrations [47], enzyme assays for AMO [48], superoxide dismutase [49,50] and cytochrome c peroxidase [51] were performed as described earlier. Glutathione reductase activity was determined using the glutathione reductase assay kit (Sigma-Aldrich). Protein samples for SDS-PAGE gels were prepared as described before [52]and separated on 10% polyacrylamide gels. Proteins were transferred to nitrocellulose membranes using the semi-dry blotting method and probed with specific polyclonal rabbit anti-AMO or anti-pyruvate carboxylase (Pyc1; loading control) antibodies. Blots were quantified using ImageJ.

### ROS measurements

ROS accumulation was measured using dihydrorhodamine 123 (DHR, Invitrogen). 10^7^ cells were harvested and stained in 50 mM potassium phosphate buffer pH=7.0 containing 20 μg/ml DHR for 30 minutes at room temperature in the dark. Cells were washed once and resuspended in the same buffer. Cells treated in same way without dye were used as controls for fluorescence background. Fluorescence signal of individual cells was captured in a FACS Aria II Cell sorter (BD Biosciences) for 10.000 events at the speed of 500-1000 events per second using a 488nm laser, 505nm long pass mirror and 525/50nm band-pass filter. FACSDiva software version 6.1.2 was used for data acquisition and analysis. The presented data represent differences in mean fluorescence between stained cells and the background.

### Fluorescence microscopy

Fluorescence microscopy images were captured using a Zeiss Axioskop 50 with a 100x 1.30 NA Plan Neofluar objective using MetaVue software and a digital camera (model 1300Y; Princeton Instruments). GFP signal was visualized with a 470/40 nm bandpass excitation filter, a 495 nm dichromatic mirror, and a 525/50-nm bandpass emission filter. ImageJ and Adobe Photoshop CS2 were used for image analysis and figure preparation. In overlay figures bright field images were false colored in blue to mark cell edges.

## SUPPLEMENTARY FIGURES


