# Supplementary material for: Peroxisomal catalase deficiency modulates yeast lifespan depending on growth conditions
Source: Aging (Albany NY). 2013 Jan 19;5(1):67–83. doi: 10.18632/aging.100519 (PMC3616232; doi:10.18632/aging.100519)
Supplement: Supplementary file 1 [file aging-05-067-s001.pdf]

46. Waterham HR, Titorenko VI, Haima P, Cregg JM, Harder W and Veenhuis M. The *Hansenula polymorpha PER1* gene is essential for peroxisome biogenesis and encodes a peroxisomal matrix protein with both carboxy- and amino-terminal targeting signals. J Cell Biol. 1994; 127:737-749.
47. Klei IJ, Harder W and Veenhuis M. Methanol metabolism in a peroxisome-deficient mutant of *Hansenula polymorpha*: a physiological study. Arch Microbiol. 1991; 156:15-23.
48. Lück H. (1963). Catalase. (New York: Academic Press).
49. Culotta VC, Klomp LWJ, Strain J, Casareno RLB, Krems B and Gitlin JD. The copper chaperone for superoxide dismutase. J Biol Chem. 1997; 272:23469-23472.
50. Flohe L and Otting F. Superoxide dismutase assays. Methods Enzymol. 1984; 105:93-104.
51. Verduyn C, Giuseppin MLF, Scheffers WA and van Dijken JP. Hydrogen peroxide metabolism in yeasts. Appl Environ Microbiol. 1988; 54 2086-2090.
52. Baerends RJ, Faber KN, Kram AM, Kiel JA, van der Klei IJ and Veenhuis M. A stretch of positively charged amino acids at the N terminus of *Hansenula polymorpha* Pex3p is involved in incorporation of the protein into the peroxisomal membrane. J Biol Chem. 2000; 275:9986-9995.
53. Sudbery PE, Gleeson MA, Veale RA, Ledeboer AM and Zoetmulder MC. *Hansenula polymorpha* as a novel yeast system for the expression of heterologous genes. Biochem Soc Trans. 1988; 16:1081-1083.
54. Nagotu S, Saraya R, Otzen M, Veenhuis M and van der Klei IJ. Peroxisome proliferation in *Hansenula polymorpha* requires Dnm1p which mediates fission but not de novo formation. Biochim Biophys Acta. 2008; 1783:760-769.
55. Nagotu S, Krikken AM, Otzen M, Kiel JAKW, Veenhuis M and Van Der Klei IJ. Peroxisome fission in *Hansenula polymorpha* requires Mdv1 and Fis1, two proteins also involved in mitochondrial fission. Traffic. 2008; 9:1471-1484.
56. Saraya R, Krikken AM, Veenhuis M and van der Klei IJ. Peroxisome reintroduction in *Hansenula polymorpha* requires Pex25 and Rho1. J Cell Biol. 2011; 193:885-900.

SUPPLEMENTARY FIGURES

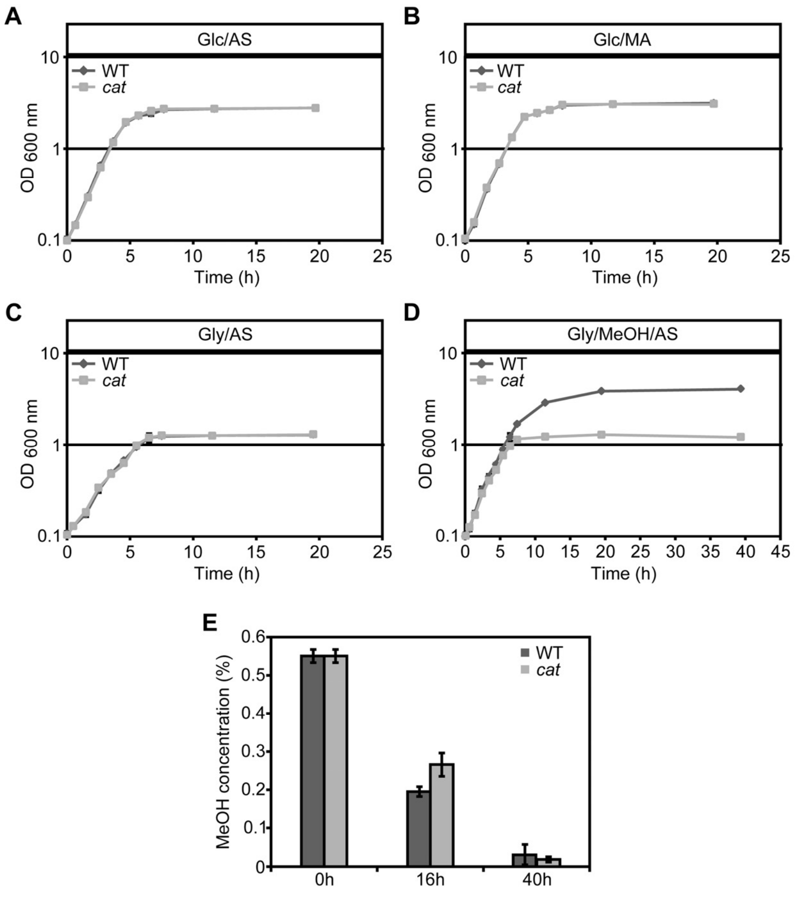

Figure S1. Growth curves of WT and *cat* cells. Cells were grown on **(A)** Glc/AS, **(B)** Glc/MA, **(C)** Gly/AS or **(D)** Gly/MeOH/AS. Optical densities are expressed as $OD_{600\ nm}$. **(E)**. Residual methanol concentrations in WT and *cat* cultures at different time points after shifting the cells to Gly/MeOH/AS. Bars indicate SD of 3 independent cultures.

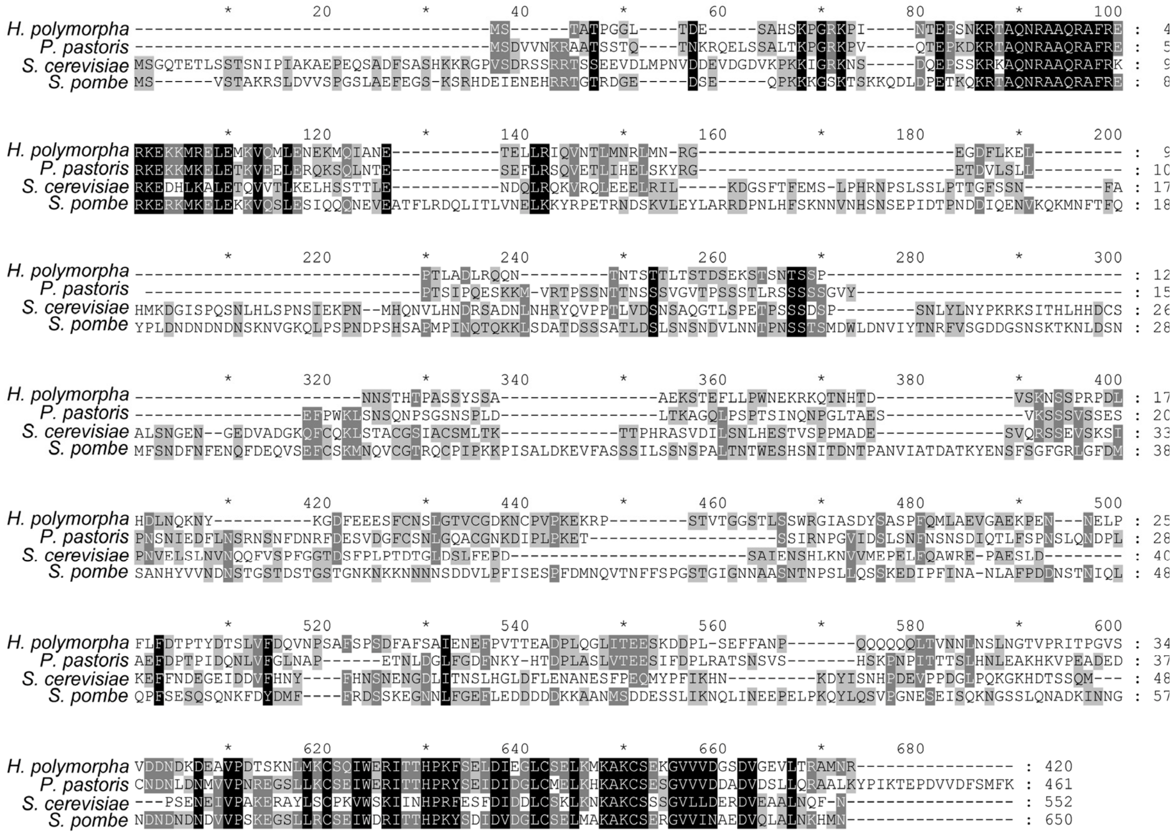

Figure S2. Sequence alignment of Yap1 homologues from different yeast species. The gene bank accession numbers are: *H. polymorpha* (EFW96135), *P. pastoris* (XP_002494040), *S. pombe* (CAB66170), *S. cerevisiae* (NP._013707).

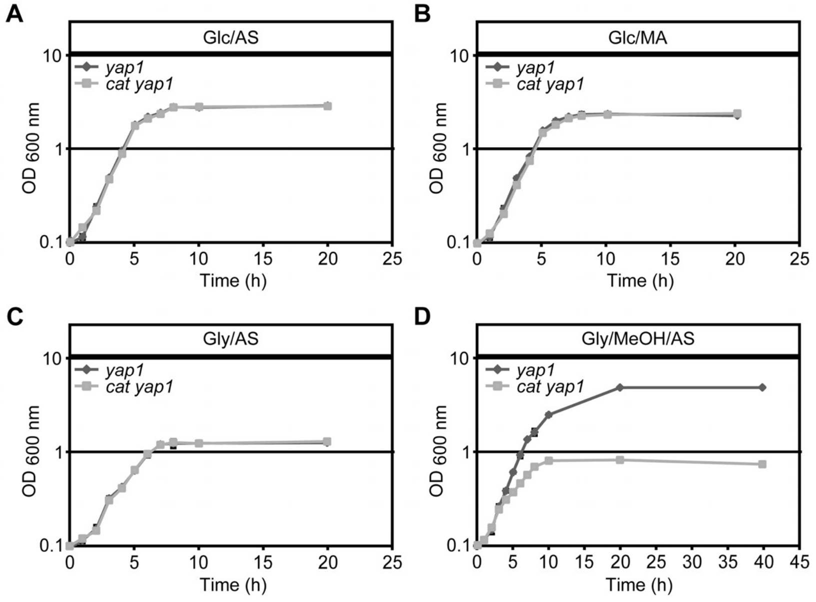

Figure S3. Growth curves of *yap1* and *cat yap1* cells. Cells were grown on (**A)** Glc/AS, (**B)** Glc/MA, (**C)** Gly/AS, **(D)** (Gly/MeOH/AS). Bars indicate SD of 2 independent cultures.
